# T-Cell Subpopulations Exhibit Distinct Recruitment Potential, Immunoregulatory Profile and Functional Characteristics in Chagas versus Idiopathic Dilated Cardiomyopathies

**DOI:** 10.3389/fcvm.2022.787423

**Published:** 2022-02-02

**Authors:** Eula G. A. Neves, Carolina C. Koh, Thaiany G. Souza-Silva, Lívia Silva Araújo Passos, Ana Carolina C. Silva, Teresiama Velikkakam, Fernanda Villani, Janete Soares Coelho, Claudia Ida Brodskyn, Andrea Teixeira, Kenneth J. Gollob, Maria do Carmo P. Nunes, Walderez O. Dutra

**Affiliations:** ^1^Department of Morphology, Cell-Cell Interactions Laboratory, Institute of Biological Sciences, Federal University of Minas Gerais, Belo Horizonte, Brazil; ^2^Brigham and Womens Hospital, Harvard University, Boston, MA, United States; ^3^Minas Gerais State University, Divinópolis, Brazil; ^4^Ezequiel Dias Foundation, Belo Horizonte, Brazil; ^5^Gonçalo Moniz Research Center, Fundação Oswaldo Cruz (FIOCRUZ), Salvador, Brazil; ^6^Rene Rachou Institute, Fundação Oswaldo Cruz (FIOCRUZ), Belo Horizonte, Brazil; ^7^Hospital Israelita Albert Einstein, São Paulo, Brazil; ^8^Instituto Nacional de Ciência e Tecnologia em Doenças Tropicais, INCT-DT, Salvador, Brazil; ^9^Graduate Program in Infectology and Tropical Medicine, School of Medicine, Federal University of Minas Gerais, Belo Horizonte, Brazil

**Keywords:** T-cells, cytokines, chemokines, inflammation, Chagas cardiomyopathy, idiopathic cardiomyopathy

## Abstract

Chronic Chagas cardiomyopathy (CCC) is one of the deadliest cardiomyopathies known and the most severe manifestation of Chagas disease, which is caused by infection with the parasite *Trypanosoma cruzi*. Idiopathic dilated cardiomyopathies (IDC) are a diverse group of inflammatory heart diseases that affect the myocardium and are clinically similar to CCC, often causing heart failure and death. While T-cells are critical for mediating cardiac pathology in CCC and IDC, the mechanisms underlying T-cell function in these cardiomyopathies are not well-defined. In this study, we sought to investigate the phenotypic and functional characteristics of T-cell subpopulations in CCC and IDC, aiming to clarify whether the inflammatory response is similar or distinct in these cardiomyopathies. We evaluated the expression of systemic cytokines, determined the sources of the different cytokines, the expression of their receptors, of cytotoxic molecules, and of molecules associated with recruitment to the heart by circulating CD4^+^, CD8^+^, and CD4-CD8- T-cells from CCC and IDC patients, using multiparameter flow cytometry combined with conventional and unsupervised machine-learning strategies. We also used an *in silico* approach to identify the expression of genes that code for key molecules related to T-cell function in hearts of patient with CCC and IDC. Our data demonstrated that CCC patients displayed a more robust systemic inflammatory cytokine production as compared to IDC. While CD8^+^ T-cells were highly activated in CCC as compared to IDC, CD4^+^ T-cells were more activated in IDC. In addition to differential expression of functional molecules, these cells also displayed distinct expression of molecules associated with recruitment to the heart. *In silico* analysis of gene transcripts in the cardiac tissue demonstrated a significant correlation between CD8 and inflammatory, cytotoxic and cardiotropic molecules in CCC transcripts, while no correlation with CD4 was observed. A positive correlation was observed between CD4 and perforin transcripts in hearts from IDC but not CCC, as compared to normal tissue. These data show a clearly distinct systemic and local cellular response in CCC and IDC, despite their similar cardiac impairment, which may contribute to identifying specific immunotherapeutic targets in these diseases.

## Introduction

Heart diseases are the leading cause of death worldwide (1). Chronic Chagas cardiomyopathy (CCC) is the most severe manifestation of Chagas disease, resulting from an intense inflammatory reaction triggered by the infection by the intracellular protozoan *Trypanosoma cruzi*, and affecting about 30% of the infected population (2). CCC is characterized by arrhythmias, thromboembolism, heart dilation and failure, and sudden death (3–5). It is considered the leading cause of non-ischemic cardiomyopathy in Latin America, and poses an economic burden of over $7 billion/year in already impoverished populations (6–8). Idiopathic dilated cardiomyopathy (IDC) comprises a heterogeneous group of clinical diseases characterized by an enlarged left ventricle with poor contractility, sharing clinical features with CCC (9–11). IDC is also the result of an inflammatory response, with evolution to congestive heart failure and death (9, 12, 13). It is one of the main causes of heart transplants in Brazil (14, 15). IDC etiology can be related to genetic and non-genetic causes, such as metabolic issues, autoimmunity or associated with previous viral infection (16, 17). Previous clinical studies have shown that CCC displays a worse prognosis when compared with cardiomyopathies of different etiologies, including IDC (18–20). This is probably related to progressive remodeling of the myocardium and consequent hypertrophy (21), in response to the intense chronic inflammation observed in CCC (22–25).

The inflammatory infiltrate present in the myocardium of CCC patients contains macrophages and B cells, but it is mainly composed of T-cells, primarily cytotoxic CD8^+^ T-cells followed by CD4^+^ T-cells (22). Although the recruitment mechanisms of these cells to the cardiac tissue of Chagas patients have not been clarified, the expression of chemotactic receptors that preferentially recruit inflammatory Th1 cells has been associated with severity of CCC (26–28). Exacerbated expression of inflammatory cytokines by circulating CD4^+^, CD8^+^ and CD4-CD8- (double negative, DN) T-cells has also been associated with CCC, as well as with worse ventricular function in these patients (29–33).

In addition to the indistinguishable clinical profile, some histopathological features observed in IDC are also shared by CCC, such as the occurrence of fibrosis and necrosis of cardiomyocytes (13). The inflammatory infiltrate associated with IDC is also mainly mononuclear, containing T and B cells, as well as macrophages (34). While CCR2 is critical for cellular recruitment in experimental models of IDC (35), it is not clear what chemokines and receptors mediate cell recruitment to human heart in IDC.

In this study, we sought to investigate phenotypic and functional features of T-cell subpopulations in CCC and IDC, aiming to clarify whether the characteristics of T-cells in the inflammation-mediated pathology is similar or distinct in these diseases. Our results showed several immunological differences related to cytokine expression, cytotoxic molecule expression and potential of recruitment to the heart in CCC vs. IDC. These systemic differences were mirrored by *in silico* analysis of transcripts found in the hearts from CCC and IDC patients, suggesting that despite the similar degree of left ventricular dysfunction and clinical resemblance, the underlying immunological mechanisms are quite distinct in CCC and IDC. Our results, in addition to providing original information regarding distinct T-cell characteristics related to disease pathology in these clinically similar diseases, point to potential targets for adjuvant immunotherapeutic approaches, potentially contributing to future clinical management of dilated cardiomyopathies.

## Patients, Materials, And Methods

### Patients

This was an observational study, with a cross-sectional analysis, including patients who were referred to a tertiary cardiology outpatient service (Hospital das Clínicas, UFMG) for management of heart failure. The patients were classified into 2 groups according to the underlying cause of cardiomyopathy. Chronic Chagas cardiomyopathy (CCC) was defined by left ventricular enlargement with systolic dysfunction in the presence of positive serologic tests for antibodies against *T. cruzi*. Idiopathic dilated cardiomyopathy (IDC) was also characterized by dilated left ventricle with systolic dysfunction, but negative serological tests for Chagas disease in the absence of abnormal loading conditions or coronary artery disease sufficient to cause global systolic impairment (36).

To analyze the *ex vivo* cellular immune profile in T lymphocyte subpopulations, peripheral blood samples of 24 individuals with CCC and 13 with IDC were collected. The demographic and clinical features of patients included in the study of T-cell responses are represented in Table 1. This table shows the similarity between the degree of cardiac involvement and clinical parameters measured in CCC and IDC patients. Except for left ventricular systolic and diastolic diameter, all other parameters were similar between groups. Plasma samples from 38 patients of the CCC group and 5 of IDC group were employed to measure circulating soluble factors. Detailed assessment, including physical examinations, electrocardiogram, chest X-rays, and echocardiogram, were performed to characterize the clinical status, as previously defined by us (3).

**Table 1:** Demographic and clinical features of patients evaluated in the study for *ex vivo* T-cell analysis.

**Characteristics**	**CCC group (*N* = 24)**	**IDC group (*N* = 17)**	***P*-value**
Age (years)	61.5 (51.5 ± 69.8)	60.0 (50.0 ± 69.0)	0.429
LVEF (%)	38.4 ± 11.6	35.3 ± 9.2	0.209
LVDD (mm)	46.3 ± 13.6	62.9 ± 9.0	**0.001**
LVSD (mm)	40.5 ± 10.0	51.0 ± 8.2	**0.004**
Left Atrial diameter (mm)	39.0 ± 10.0	44.4 ± 6.6	0.08
SPAP (mmHg)	34.1 ± 4.5	33.8 ± 13.0	0.471
Heart rate (bpm)	60.0 (54.8 ± 61.5)	68.0 (57.0 ± 80.0)	0.148
A (cm/s)	63.0 (36.2 ± 78.8)	34 (30.0 ± 89.5)	0.347
E (cm/s)	66.0 ± 13.5	56.8 ± 4.6	0.11
DT (ms)	199.5 ± 65.1	205.0 ± 67.4	0.443

*Data are presented as mean ± SD or median and interquartile range. Sex Variable were presented by percentage (%). LVEF, left ventricular ejection fraction; LVDD, left ventricular end-diastolic diameter; LVSD, left ventricular end-systolic diameter; SPAP, systolic pulmonary artery pressure; A, late diastolic trans mitral flow velocity; E, early diastolic trans mitral flow velocity; DT, deceleration time. The p-values in bold indicates statistically significant differences between CCC and IDC*.

All individuals went through cardiological clinical follow-up at the Hospital das Clínicas of Universidade Federal de Minas Gerais (UFMG) and accepted to participate voluntarily in the research. All were informed about the objectives of our study and signed the Informed Consent Form. This study was approved by the Comitê de Ética em Pesquisa of Universidade Federal de Minas Gerais (COEP-UFMG – ETIC006/05) and Comissão Nacional de Ética em Pesquisa (CONEP no. 2.809.859) and conformed to the ethical guidelines of the 1975 Declaration of Helsinki.

### Blood Sampling and Collection of Peripheral Blood Mononuclear Cells (PBMC) and Plasma

Peripheral blood samples were collected by venipuncture in tubes containing sodium heparin (Vacutainer, Becton Dickinson, San Jose, CA, USA). PBMC were isolated by differential centrifugation at 600 g for 40 min, using Ficoll-Paque PLUS (GE Healthcare, Sweeden), as previously done by us (37). Plasma was retrieved and PBMC were washed with phosphate buffered saline three times, and resuspended in RPMI 1640 medium (Thermo Fisher Scientific, Waltham, US), supplemented with 5% inactivated human serum (Thermo Fisher Scientific, Waltham, US), 1% antibiotic (penicillin, 200 U/mL; and streptomycin, 0.1 mg/mL, -Thermo Fisher Scientific, Waltham, US) and 1 mM of L-glutamine (Sigma-Aldrich, St. Louis, US) at a concentration of 1 × 10^7^ cells/ml. The cells were kept in a CO_2_ incubator at 37°C for 18 h, and 1 μg/mL of Brefeldin A (Biolegend, San Diego, CA) was added in the last 4 h to prevent cytokine secretion.

To assess the frequency of TNF in CD8^+^ T lymphocytes and for the correlation analysis with the frequency of IFN-gamma in CD4^+^ and CD8^+^ T lymphocytes, PBMC from 7 CCC patients and 8 IDC patients were stimulated with PMA (1 ng/mL, Sigma-Aldrich, St. Louis, US) and Ionomycin (500 ng/mL, Sigma-Aldrich, St. Louis, US), under the same conditions mentioned above.

### Measurement of Soluble Cytokines and Chemokines

Plasma obtained from peripheral blood as mentioned above was used to measure the following molecules: cytokines (IL1-Ra, IL-2, IL-6, IL-7, IL-10, IL-15, IL-17) and chemokines (CCL3, CCL4, CCL5) using the Bio-Plex ProTM Human Cytokine Standard 27-plex Kit (Bio-Rad—Hercules, CA, USA). Experiments were performed according to the manufacturer's instructions. The data were acquired by the Bio-Plex 200 instrument equipped with the Manager software, and the results were expressed as the mean fluorescence intensity (MFI) after background subtraction.

### Flow Cytometric Analysis

PBMC (2 × 10^5^/tube) were stained for 30 min at 4°C with a combination of anti-cell surface molecules monoclonal antibodies conjugated to fluorochromes. Anti-CD4 PercpCy5 (clone A161A1), CD8 APCcy7 (clone SK1), TCR alpha-beta FITC (Clone IP-26) and TCR gamma-delta BV421 (clone B1) were used for phenotypic identification of T-cell subpopulations; anti-TNFR1 APC (Clone W15099A) and anti-IL10R PE (clone 3F9), for evaluation of immunoregulatory cytokines receptors; anti-CCR5 BV510 (clone J418F1), anti-CXCR3 pecy7 (clone G025H7), and anti-CCR4 BV510 (clone L291H4), for analysis of chemokine receptor expression.

After the incubation period, cells were washed twice by centrifugation (10 min, 4°C, 600 g) with PBS containing 1% BSA and fixed with 2% paraformaldehyde solution for 20 min. After total removal of the fixing solution by centrifugation and washing with PBS, the cells were permeabilized for 15 min with 0.5% saponin and then submitted to intracellular staining with anti-IFN-gamma BV510 (clone 4S.B3), anti-TNF BV510 (clone MAb11), anti-IL-10 APC, (clone JES3-19F1), anti-IL-17 BV510 (clone (BL168), anti-granzyme A PE (CB9), anti-perforin PE (clone B-D48), anti-Eomes APC (clone # 644730) and anti-cMET APC (clone # 95106). All combinations employed antibodies labeled with different fluorochromes. Anti-Eomes and anti-cMET antibodies were from R&D Systems (Minneapolis), and all others were from Biolegend (San Diego, CA, USA).

Subsequently, the cells were washed twice with a 0.5% saponin solution and resuspended in PBS for acquisition in a FACSCanto II flow cytometer (Becton & Dickinson, San Jose, CA, USA). A minimum of 100,000 gated events was acquired and analyzed using the software Flowjo (Ashland, Oregon-US), employing supervised analysis and unsupervised machine-learning strategies. We analyzed forward scatter area (FSC-A) vs. forward scatter height (FSC-H) to remove doublets. Lymphocytes were selected based on FSC-A vs. side scatter area (SSC-A). The analysis strategy for evaluating the different T-cell subpopulations is represented in Figure 2A.

### *In silico* Analysis of mRNA Expression Profile

Data containing the gene expression profile by microarray analysis were obtained from the Gene Expression Omnibus (GEO) database (http://www.ncbi.nlm.nih.gov/geo/). A total of 17 samples of the human left ventricular free wall heart tissue were available for comparison between CCC (*n* = 10) and healthy donors (*n* = 7), under the Gene Expression Omnibus accession number GSE84796; files were selected to compare the gene expression profile of CD4, CD8, IFN-gamma, IL-10, Il-17, granzyme A, perforin, CCR5, CCL3, CCL4, CCL5, Eomes, and HGF; data Evaluated by SurePrint G3 Human GeneExpression v1 8x60K Arrays (Agilent Technologies, Les Ulis, França) (38). Additionally, through accession number GPL2041, files corresponding to gene transcripts of CD4, CD8, Granzyme A, Perforin, CCR5, CCL5, and Eomes were selected from the analysis of 7 samples of the left ventricular anterior free wall from patients with IDC and 8 of healthy donors (GeneChip^®^ Human Gene 1.0 array-Affymetrix) (39). Gene expression profile data from both studies were represented using mRNA fluorescence intensity.

### Protein-Protein Interaction Network and Enriched Pathways Analysis

The network was constructed using NetworkAnalyst.ca. through direct relationships between proteins and altered soluble factors in CCC and IDC. Pairwise correlation prediction was determined based on IMEx database. Resulting high-scoring genes were used to identify hub genes. Enriched pathway analysis emerging as a result of interconnections in the network were generated using Kyoto Encyclopedia of Genes and Genomes (KEGG).

### Statistical Analysis

To assess the systemic profile of soluble factors, the phenotypic and functional cellular profile of T-cell subpopulations among cardiomyopathies, and comparative analysis of the *in silico* study, the data were analyzed using the GraphPad Prism 8 software (GraphPad Software, La Jolla—CA, USA). After evaluating the Gaussian distribution using the Shapiro-Wilk test, the parametric data were analyzed using the unpaired *t*-test end non-parametric data were submitted to the Mann-Whitney test. The data were represented in the graphs using minimum and maximum values. Correlation analysis of the plasma profile of the chemokine CCL4 with its ligand CCR5 in CD4^+^, CD8^+^ and gamma-delta^+^ DN T cells, as well as the evaluation of association the % expression of IFN-gamma with cMET^+^ CCR5^+^ and CXCR3^+^ CCR4^+^ cells in different cardiomyopathies, was performed using Pearson's test for parametric data and Spearman's test for non-parametric data.

To assess a possible pattern of differentiation between groups, using data from soluble cytokines, we built a representative heat map analysis using the Clustvis software, which uses the R-version 0.7.7 package, where it is possible to identify the cluster between the samples and define the homogeneity or heterogeneity in the distribution of data between the groups. Rows and columns are grouped using the correlation distance and the mean link. Additionally, principal component analysis (PCA) was performed, in which the X- and Y-axes show the % of the total variance. Prediction ellipses indicate a probability of 0.95 that a new observation will fall inside the ellipse (40).

For the qualitative representation of the cellular immune profile, analysis of the t-distributed stochastic neighbor-embedding (t-SNE) algorithm was performed, a tool that allows the visualization of multidimensional data in 2 dimensions (t-SNE1 and t-SNE2) (41). Then, the t-SNE analysis was generated using the Barnes-Hut algorithm with 1,000 interactions and a perplexity parameter of 30. The molecules selected for this analysis were CD4, CD8, TCR gamma-delta, CXCR3, CCR4, and cMET.

## Results

### The Levels of Inflammatory, Proliferative, and Regulatory Cytokines Are Elevated in Plasma of CCC, as Compared to IDC Patients

**Figure 1:**
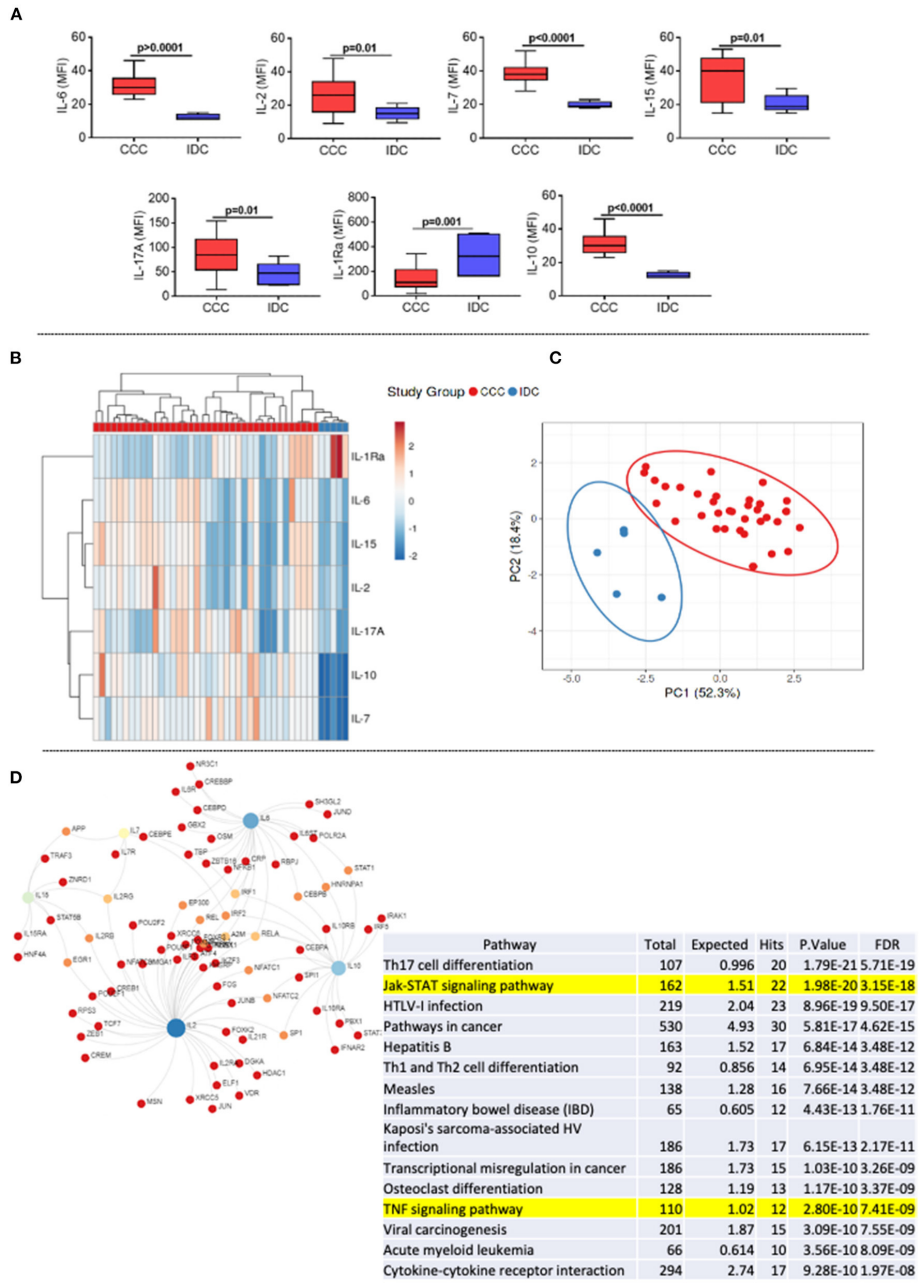
Comparative analysis of plasma cytokines levels between the study groups. **(A)** Comparative analysis of plasma cytokine levels between patients with Chronic Chagas cardiomyopathy (CCC, *n* = 38) and Idiopathic cardiomyopathy (IDC, *n* = 5). Plasma soluble cytokine levels were measured using the Bio-Plex ProTM Human Cytokine Standard 27-plex Kit, and results are expressed in MFI, as described in Materials and Methods. Graphs are expressed as boxplots, with the minimum and maximum values indicated. *p*-values < 0.05 were considered statically significant and are shown in the Figure. **(B)** Representative heat map analysis of cytokine plasma levels. Both rows and columns are grouped using correlation distance and mean link. In the color gradient bar, blue indicates lower plasma levels, while red indicates higher levels. Vertical lines represent each sample evaluated, and horizontal lines represent each molecule in the study. **(C)** Principal component analyses (PCA) between measured cytokines and evaluated study groups; the X and Y axes show % of the total variance. Prediction ellipses indicate with a probability of 0.95 that a new observation will fall within the ellipse. **(D)** Network and enriched pathway analysis in CCC. Protein-protein network interactions were performed considering the molecules altered in CCC as compared to IDC. Blue and light green represent rub nodes. Different colors distinguish nodes with different numbers of interactions. Bottom table shows a pathway enrichment analysis derived from protein-protein interactions based on Kyoto Encyclopedia of Genes and Genomes (KEGG) algorithm, showing the top 20 hits for CCC with lowest false discovery rates (FDR). T-cell signaling and TNF signaling pathways are highlighted.

First, we assessed the systemic soluble cytokine profile in plasma samples of patients with different cardiomyopathies. Our data showed an increase in cytokines with an inflammatory profile (IL-6), proliferative (IL-2, IL-7, IL-15), and regulatory (IL-10 and IL-17) in the CCC group compared to IDC (Figure 1A). On the other hand, the level of the IL-1 receptor antagonist (IL-1RA) were higher in IDC than CCC (Figure 1A). Heatmap analysis showed clear segregation between groups, with a strong correlation between IL-15 and IL-2 and IL-10 and IL-7 (Figure 1B). PCA analysis reinforced the distinct soluble cytokine profile between cardiomyopathies, shown by the prediction ellipses, with a total variation of 70.7%, with 52.3% on the X-axis and 18.4% on the Y-axis (Figure 1C). Network analysis using the molecules that were altered in CCC (Figure 1A) as compared to IDC shows the inflammatory cytokine IL-6, the proliferative cytokines IL-15 and IL-2 and the modulatory cytokine IL-10 as central nodes, with high centrality (Figure 1D). Enriched pathway analysis emerging as a result of interconnections in the network generated by KEGG, showed a predominance of inflammatory networks in CCC. Of note, T-cell signaling and TNF signaling pathways emerge within the first 20 pathways with lowest false discovery rate (FDR, adjusted *p*-value) in CCC (Figure 1D, yellow mark).

### Inflammatory CD8^+^ T-Cells Are More Frequent in CCC Patients, While Inflammatory CD4^+^ T-Cells Are Associated With IDC

**Figure 2:**
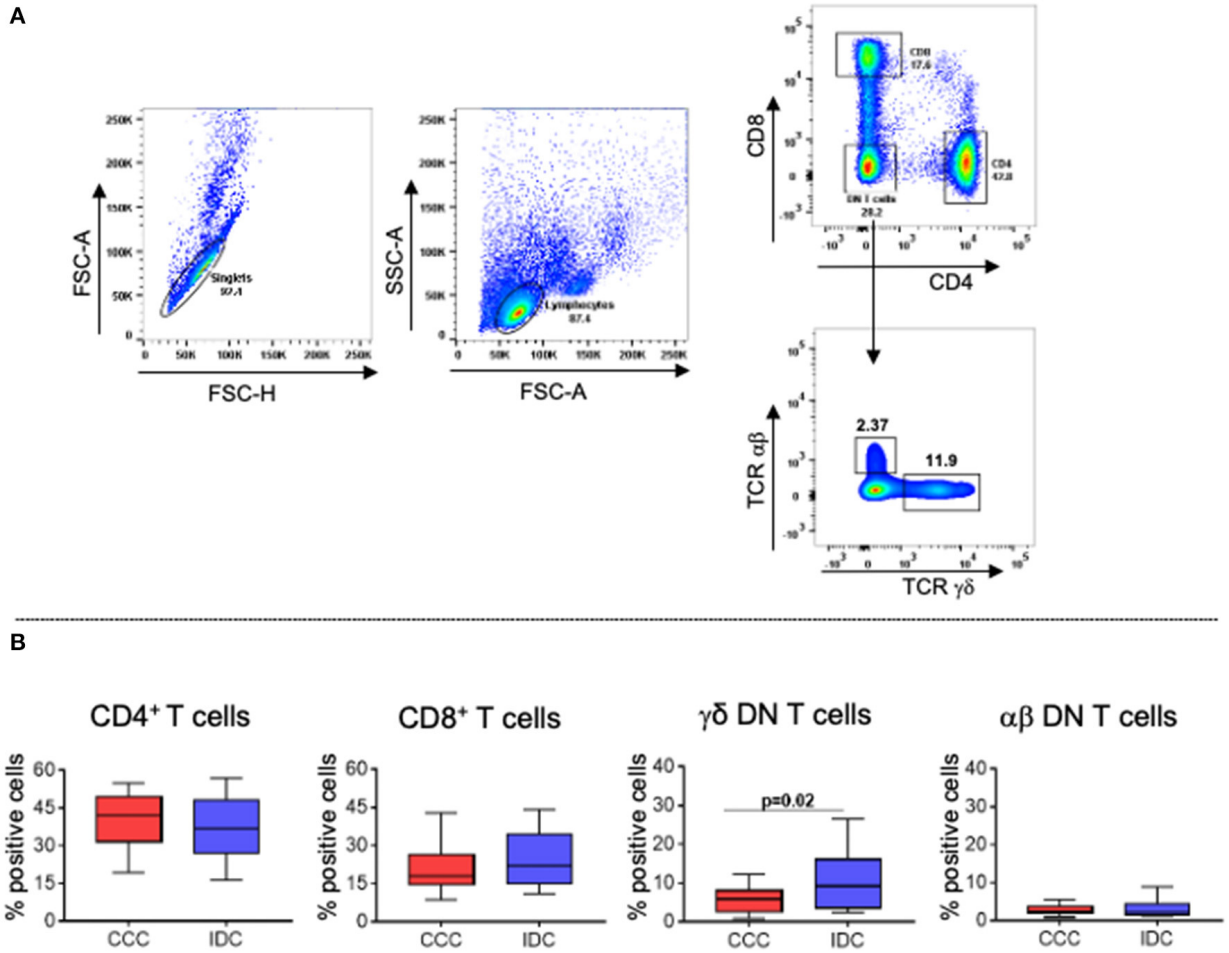
Frequency of different T lymphocyte subpopulations in peripheral blood of patients with chronic Chagas cardiomyopathy and idiopathic cardiomyopathy. **(A)** Representative dot plots of the analysis strategy used in the study: To remove doublets, the parameters FSC-A × FSC-H were used; lymphocytes were selected according to size (FSC-A) and granularity (SSC-A) parameters. For phenotypic separation of cell subpopulations, CD8 and CD4 surface markers were selected, followed by selection of CD4−CD8− cells to identify gamma−delta^+^ and alpha−beta^+^ DN T cells. **(B)** Frequency of CD4^+^ T cells, CD8^+^ T cells, gamma−delta^+^ DN T cells (CCC, n = 17; IDC, n = 13), and alpha−beta^+^ DN T cells (CCC, n = 10; IDC, n = 10). Graphs are expressed as boxplots, with the minimum and maximum values indicated.

To evaluate cellular responses, we first determined the overall frequency of circulating T-cell subpopulations, evaluating the frequencies of CD4^+^, CD8^+^, TCRgamma-delta^+^ and TCRalpha-beta^+^ DN T-cells in the different cardiomyopathies. Figure 2A shows the analysis strategy employed. Our data showed that the frequencies of CD4^+^, CD8^+^ and alpha-beta^+^ DN T-cells were similar between the groups (Figure 2B). However, we observed higher frequency in DN T-cells expressing the TCR gamma-delta in IDC compared to CCC patients (Figure 2B).

**Figure 3:**
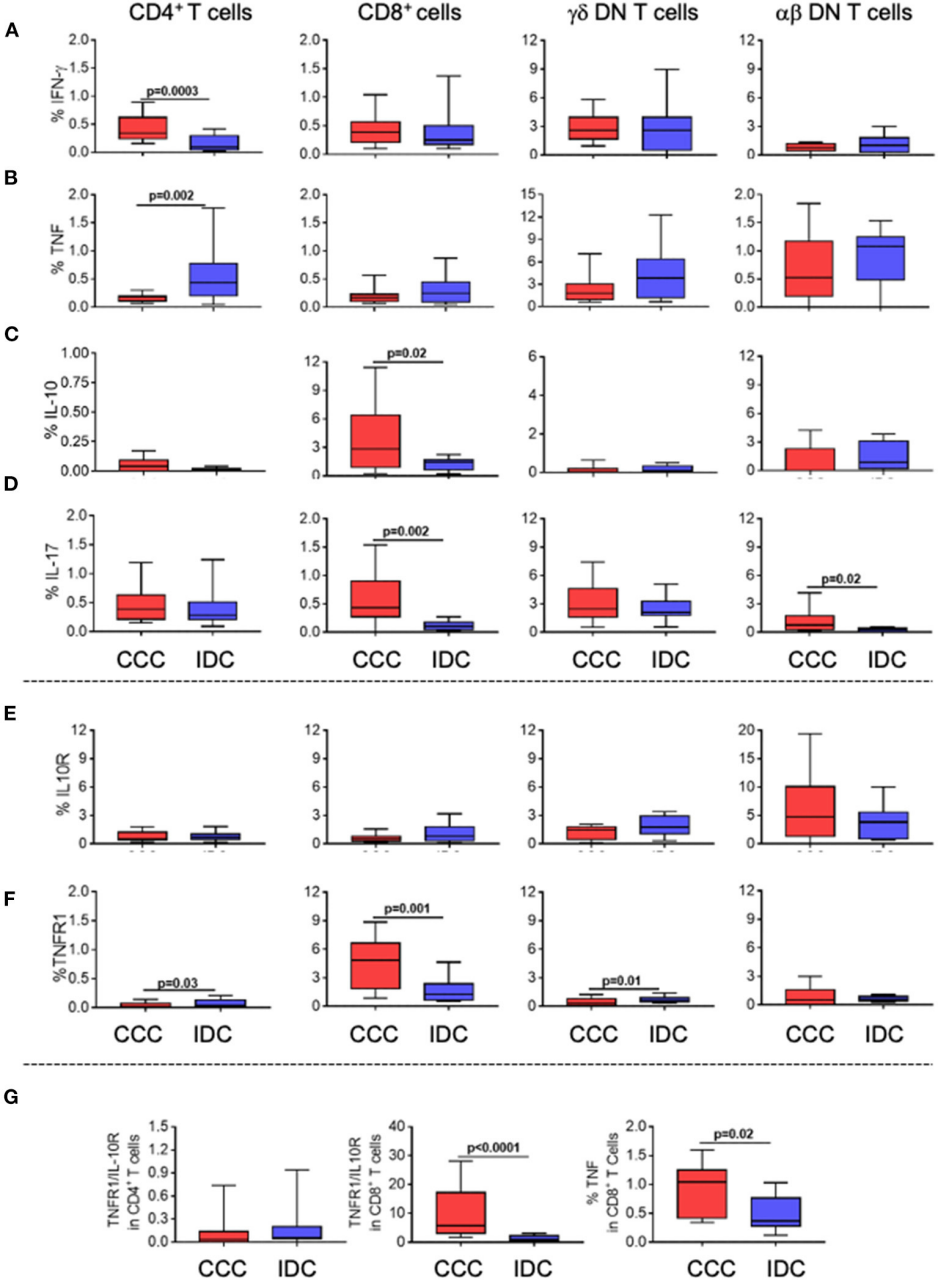
Cellular immune profile of cytokines and immunoregulatory receptors in T-cell subpopulations in different cardiomyopathies. Frequency of expression of cytokines and immunoregulatory receptors **(A)** IFN-gamma, **(B)** TNF, **(C)** IL-10 **(D)** IL-17 **(E)** IL-10R, **(F)** TNF-R1. The analysis was performed as described in the materials and methods for comparison between the different groups in CD4^+^, CD8^+^, gamma-delta^+^ (CCC, *n* = 17, IDC, *n* = 13) and alpha-beta^+^ DN T cells (CCC, *n* = 10, IDC, *n* = 10). **(G)** analysis of the ratio of TNFR1+/IL10R+ cells in CD4^+^ and CD8^+^ T cells; TNF expression in CD8^+^ T cells stimulated with PMA/Ionomycin (CCC, *n* = 7; IDC, *n* = 8). Values of *p* < 0.05 were considered statistically significant. Graphs are expressed as boxplots, with the minimum and maximum values indicated.

To assess the functional profile of the different T-cell subpopulations, we evaluated the expression of the inflammatory cytokines IFN-gamma and TNF, as well as of the regulatory cytokine IL-10, and of IL-17. We observed an increase in the frequency of IFN-gamma (Figure 3A) in CD4^+^ T-cells in the CCC group compared to IDC, while TNF was increased in CD4^+^ T-cells from IDC as compared to CCC (Figure 3B). The frequency of CD8^+^IL-10^+^ T-cells was increased in CCC as compared to IDC (Figure 3C). IL-17 expression was increased in CD8^+^ and alpha-beta^+^ DN T-cells from CCC as compared to IDC (Figure 3D).

Analysis of TNF receptor 1 (TNFR1) and IL-10 receptor (IL-10R) expression showed that, while there was no difference in the expression of IL-10R by the different T-cell subpopulations (Figure 3E), TNFR1 was upregulated in CD4^+^ T-cells and TCRgamma-delta^+^ DN T-cells from IDC than CCC, and higher in CD8^+^ cells from CCC compared to IDC (Figure 3F). We observed a higher TNFR1/IL-10R ratio in CD8^+^ T-cells from CCC as compared to IDC, and a tendency of increased TNFR1/IL-10R ratio in CD4^+^ cells in IDC (Figure 3G). In addition, we observed a higher frequency of CD8^+^TNF^+^ T-cells in CCC as compared to IDC after *in vitro* stimulation (Figure 3G). Together, these data suggest that CD8^+^ T-cells and CD4^+^ T-cells from CCC and IDC, respectively, display a predominantly inflammatory profile.

**Figure 4:**
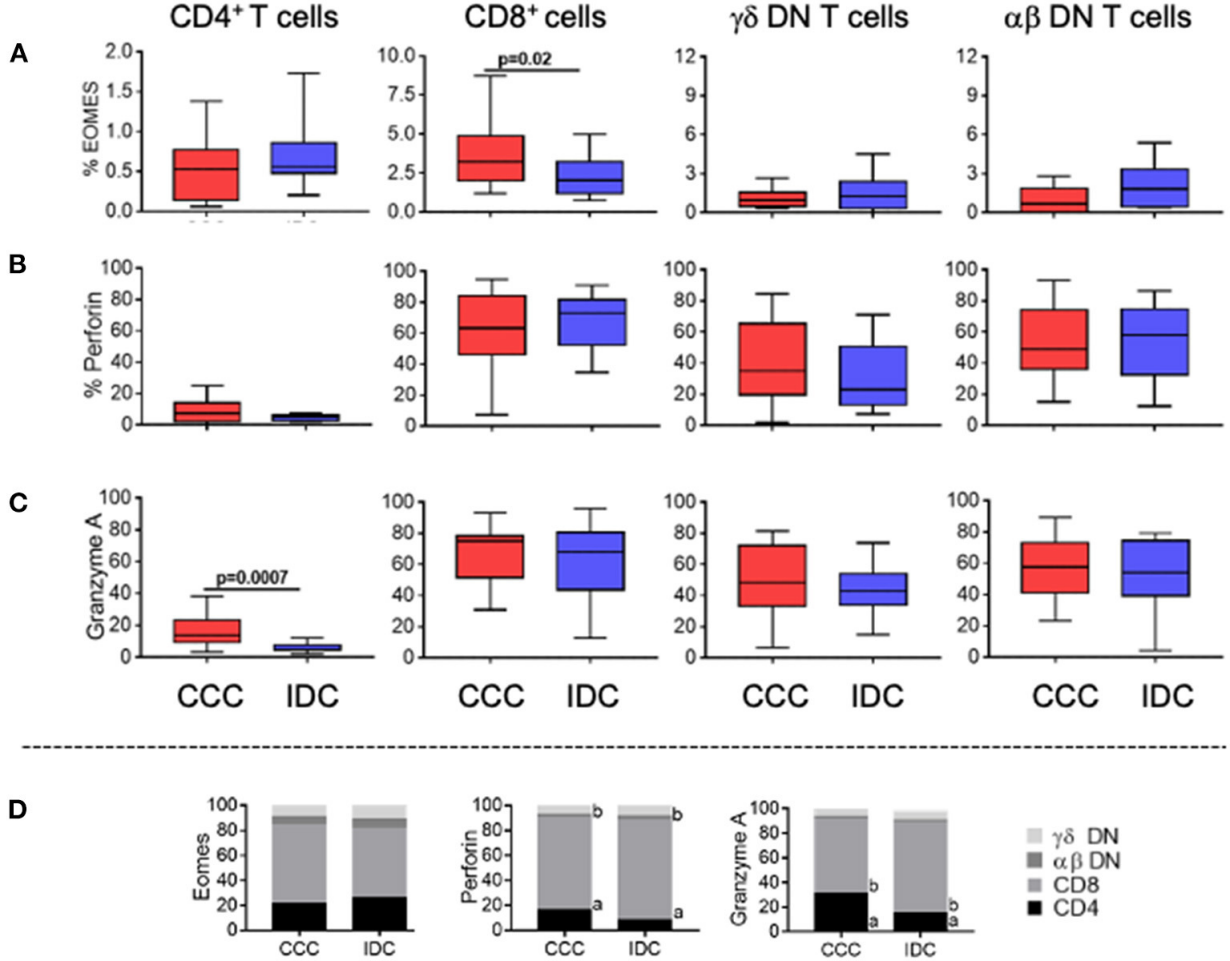
Expression of cytotoxic molecules by the different T-cell subpopulations. Frequency of expression of molecules associated with cytotoxic function **(A)** Eomes, **(B)** perforin and **(C)** granzyme A are shown. Graphs are expressed as boxplots, with the minimum and maximum values indicated. **(D)** Contribution of different cell subpopulations to the expression of different cytotoxic molecules. The analysis was performed as described in the materials and methods for comparison between the different groups in CD4^+^, CD8^+^, gamma-delta^+^ DN T cells (CCC, *n* = 17, IDC, *n* = 13) and alpha-beta^+^ DN T cells (CCC, *n* = 10, IDC, *n* = 10). Values of *p* < 0.05 were considered statistically significant.

To determine the cytotoxic potential of the different T-cell subpopulations, we evaluated the frequency of expression of Eomes, perforin, and granzyme A. We observed a significant higher frequency of Eomes only in CD8^+^ T-cells in the CCC group compared to IDC (Figure 4A). No differences were observed in perforin expression comparing CCC and IDC (Figure 4B). Expression of granzyme A was increase in CD4^+^ T-cells in CCC patients compared to IDC (Figure 4C).

We also verified the percentage contribution of the different T-cell subpopulations to the expression of the different cytotoxic molecules. It is quite clear that CD8^+^ cells are the main expressors of cytotoxic molecules, as compared to the other T-cell subpopulations analyzed in both CCC and IDC (Figure 4D). In addition to CD8^+^ T cells, DN TCR alpha beta T cells showed a statistically significant increase in the contribution to perforin expression in IDC compared to CCC, emerging as an important source of perforin expression in IDC (Figure 4D).

### Analysis of Chemokine and Chemokine Receptor Expression Reveals Distinct Recruitment Potential of T-Cell Subpopulations in the Different Cardiomyopathies

**Figure 5:**
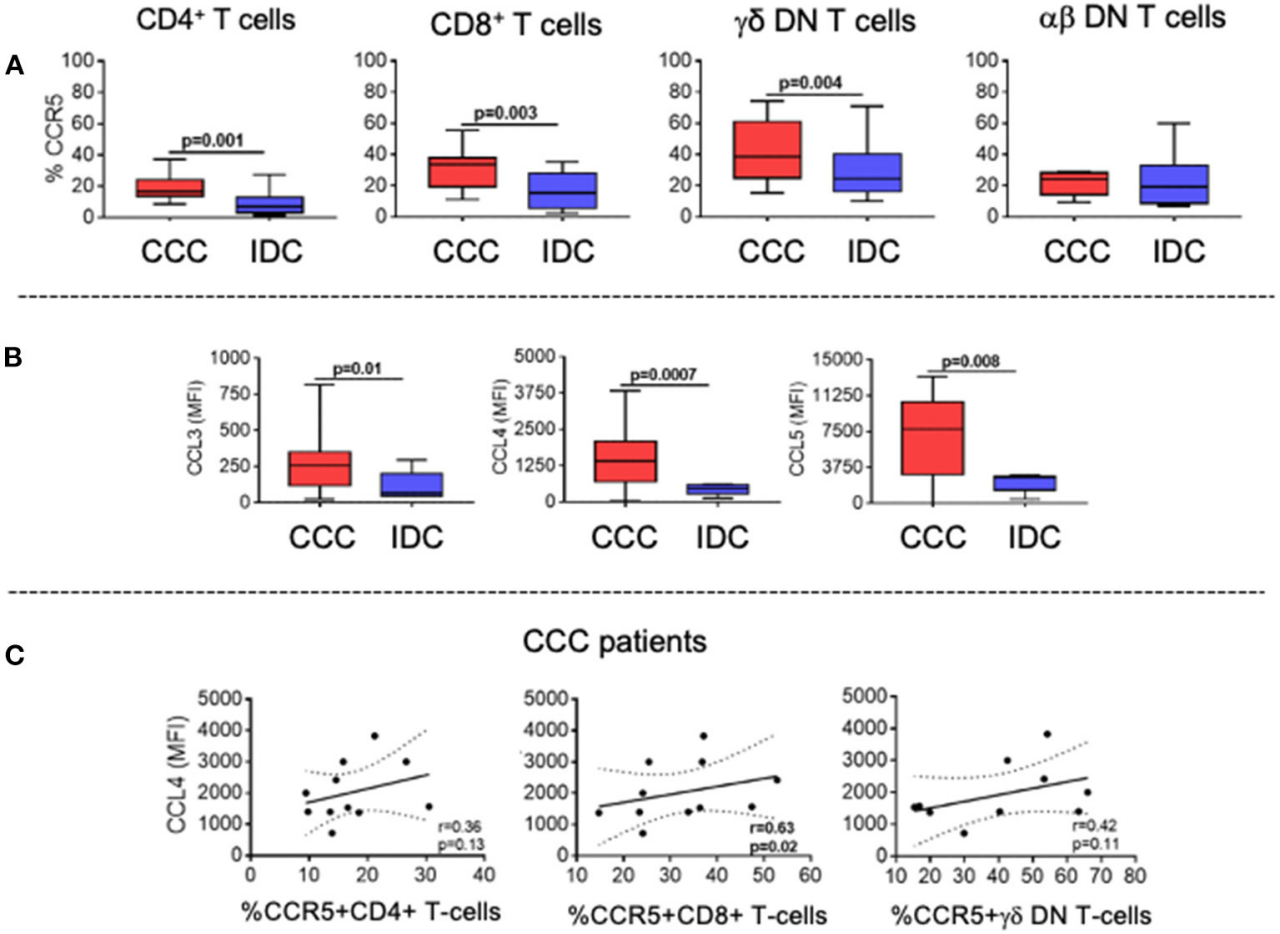
Expression of CCR5 by the different T-cell subpopulations and of its soluble chemokine ligands CCL3, CCL4, CCL5. **(A)** Evaluation of the frequency of expression of CCR5 by CD4^+^, CD8^+^, gamma-delta^+^ DN T cells (CCC, *n* = 17, IDC, *n* = 13) and alpha-beta+ DN T cells (CCC, *n* = 10, IDC, *n* = 10). The analysis was performed as described in the materials and methods for comparison between the different groups. **(B)** Plasma levels of soluble chemokines (CCL3, CCL4, CCL5) in samples from patients with Chronic Chagas cardiomyopathy (CCC, *n* = 38) and idiopathic cardiomyopathy (IDC, *n* = 5). Graphs are expressed as boxplots, with the minimum and maximum values indicated. **(C)** Correlation analysis between plasma levels of CCL4 and the frequency of expression of CCR5 in CD4^+^, CD8^+^ and gamma-delta^+^ DN T cells in CCC group. Parametric data were analyzed using Pearson's correlation test and non-parametric data using Spearman's test. Values of *p* < 0.05 were considered statistically significant.

The expression of the chemotactic receptor CCR5, which has been associated with CCC, was significantly higher in CD4^+^, CD8^+^, and TCRgamma-delta^+^ DN T-cells in the CCC group as compared to IDC (Figure 5A). We also measured the systemic expression of the chemokines CCL3, CCL4, and CCL5, which are ligands for CCR5 and associated with cellular recruitment to inflammatory sites. Our data showed increased plasma levels in these chemokines in the CCC group as compared to IDC (Figure 5B).

To determine the association between the CD4^+^, CD8^+^, and TCRgamma-delta^+^ DN T-cells expressing CCR5 with plasma levels of the chemokine ligands, we performed a correlation analysis between these molecules in the CCC group. While no correlation was observed between the cells expressing CCR5 and CCL3 or CCL5, we observed a positive and statistically significant correlation between expression of CCL4 and the frequency of CD8^+^CCR5^+^, but not with CD4^+^ nor gamma-delta^+^ DN T-cells (Figure 5C). These data indicate an association between CCL4 expression and CD8^+^CCR5^+^ T-cells in CCC.

**Figure 6:**
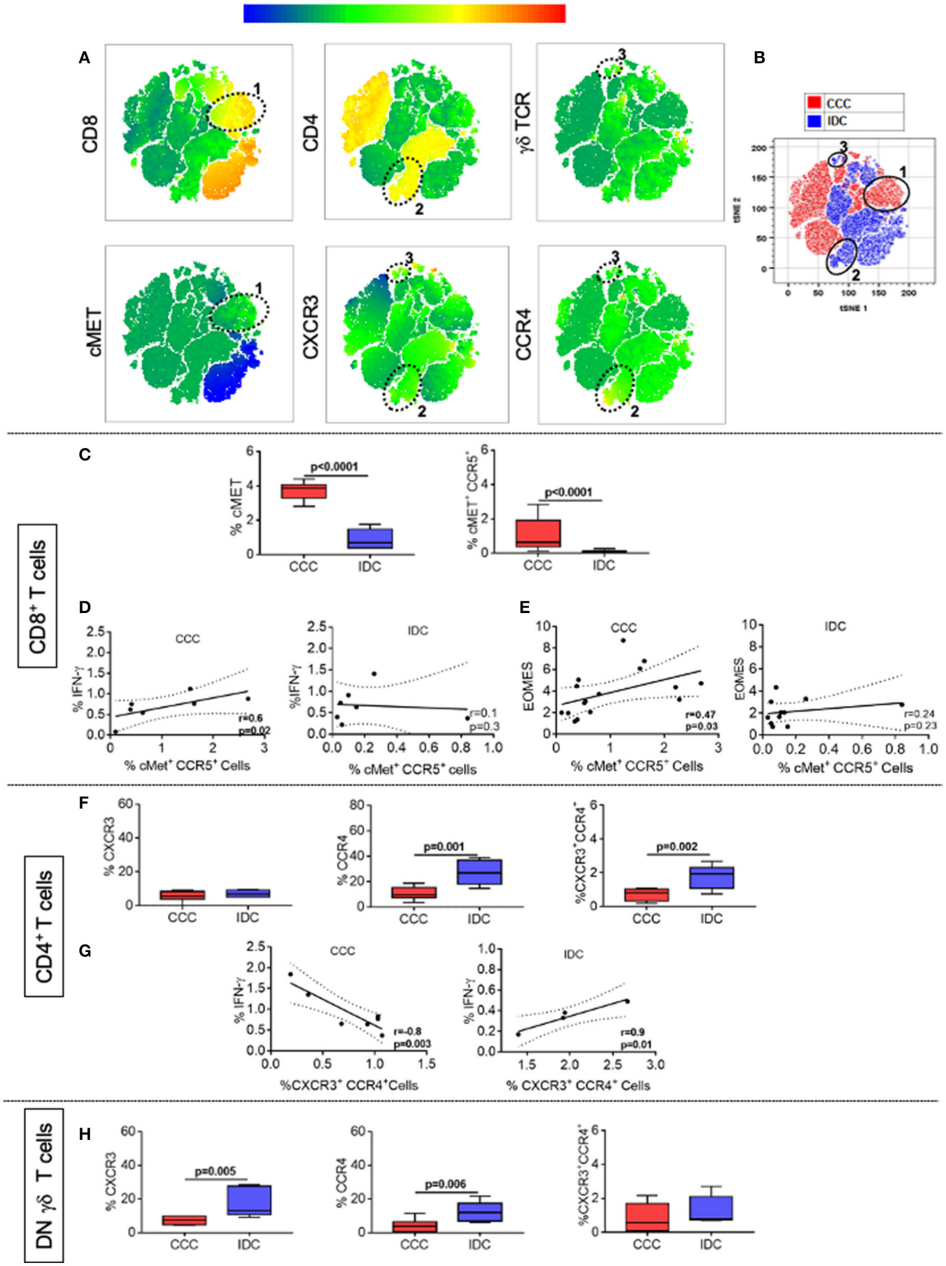
CCR4 and CXCR3 expression reveals distinct recruitment potential between cardiomyopathies. **(A)** Representation of t-distributed stochastic neighbor embedding (t-SNE) using the expression density of CD4, CD8, cMET, TCR gamma-delta, CXCR3, and CCR4, with emphasis on clusters 1, 2, and 3 shown by the ellipses. Cluster 1 shows the colocalization between cMET and CD8 expression, while clusters 2 and 3 demonstrate the expression of CXCR3 and CCR4 by CD4^+^ and gamma-delta^+^ DN T cells, respectively. **(B)** tSNE generated showing the stratification between the CCC (red) and IDC (blue) groups after overlaping the islands formed by the algorithm. **(C)** Frequency (%) of cMET^+^ and co-expression of cMET^+^CCR5^+^ in CD8^+^ T cells (CCC, *n* = 17, IDC, *n* = 13). **(D)** Correlation analysis between the frequency of CD8^+^ IFN-gamma^+^ cells stimulated with PMA/Ionomycin (CCC, *n* = 7; IDC, *n* = 8) and CD8^+^cMET^+^ CCR5^+^ cells. **(E)** Correlation analysis between the frequency of CD8^+^Eomes^+^ cells and CD8^+^cMET^+^CCR5^+^ cells. **(F)** Frequency (%) of CXCR3, CCR4 and co-expression of CXCR3^+^ CCR4^+^ in CD4^+^ T cells (CCC, *n* = 8; IDC, *n* = 5). **(G)** Correlation analysis between the frequency of CD4^+^ IFN-gamma^+^ cells stimulated with PMA/Ionomycin (CCC, *n* = 7, IDC, *n* = 5) and CD4^+^ CXCR3^+^ CCR4^+^ cells. **(H)** Frequency of CXCR3, CCR4 and co-expression of CXCR3^+^ CCR4^+^ in gamma-delta^+^ DN T cells (CCC, *n* = 8; IDC, *n* = 5). The analysis was performed as described in the materials and methods for comparison between the different groups. Graphs are expressed as boxplots, with the minimum and maximum values indicated. Values of *p* < 0.05 were considered statistically significant.

We also evaluated the frequency of expression of the chemokine receptors CXCR3, CCR4, and the cardiotropic molecule cMET in CD4^+^, CD8^+^, and TCRgamma-delta^+^ DN T-cells, using multiparameter flow cytometry, combined with conventional and unsupervised machine-learning strategies. Through clustering by similarity using unsupervised analysis, we found different population clusters, which revealed differences in the expression density of the mentioned molecules. We identified clusters 1 (Figure 6A), distributed within the CCC group (red color, Figure 6B), and clusters 2 and 3 (Figure 6A), predominantly located in the IDC group (blue color, Figure 6B). Cluster 1 shows the colocalization between cMET and CD8 expression, while clusters 2 and 3 demonstrate co-localization of CXCR3 and CCR4 in CD4^+^ T-cells and gamma-delta DN T-cells, respectively. Conventional quantitative analysis confirmed the data observed by tSNE, showing a significant higher expression of CD8^+^cMET^+^ T-cells, as well as CD8^+^cMET^+^CCR5^+^ T-cells in the CCC group compared to IDC (Figure 6C). A positive correlative analysis between the frequency of CD8^+^CCR5^+^cMET^+^ T-cells and the inflammatory cytokine IFN-gamma or Eomes was observed in CCC but not IDC (Figures 6D,E).

As observed in the unsupervised qualitative analysis, the frequency of CCR4 and the co-expression of CXCR3 and CCR4 were higher in CD4^+^ T-cells in IDC when compared to the CCC (Figure 6F). A positive correlation between the frequency of CXCR3^+^CCR4^+^ CD4^+^ T-cells and the inflammatory cytokine IFN-gamma was observed in IDC but not CCC (Figure 6G). In addition, the expression of CXCR3 and CCR4 were higher in TCRgamma-delta^+^ DN T-cells in IDC than CCC (Figure 6H). These data support the hypothesis of a distinct cell recruitment potential of activated T-cells populations in the different cardiomyopathies.

We performed a combined analysis of the parameters that were statistically significant comparing between groups, including cytokine, cytotoxic molecule, and chemotactic receptor expression by CD4^+^ and CD8^+^ T-cells in IDC and CCC, respectively. The Clustvis algorithm showed a distribution that generated a cellular immune signature profile that segregated the CCC and IDC groups, emphasizing the cellular immunological differences between the cardiomyopathies (Supplementary Figure 1).

### *In silico* Analysis Reveals Inflammatory and Cytotoxic Potential by CD8^+^ T-Cells in the Heart of Patients With CCC

**Figure 7:**
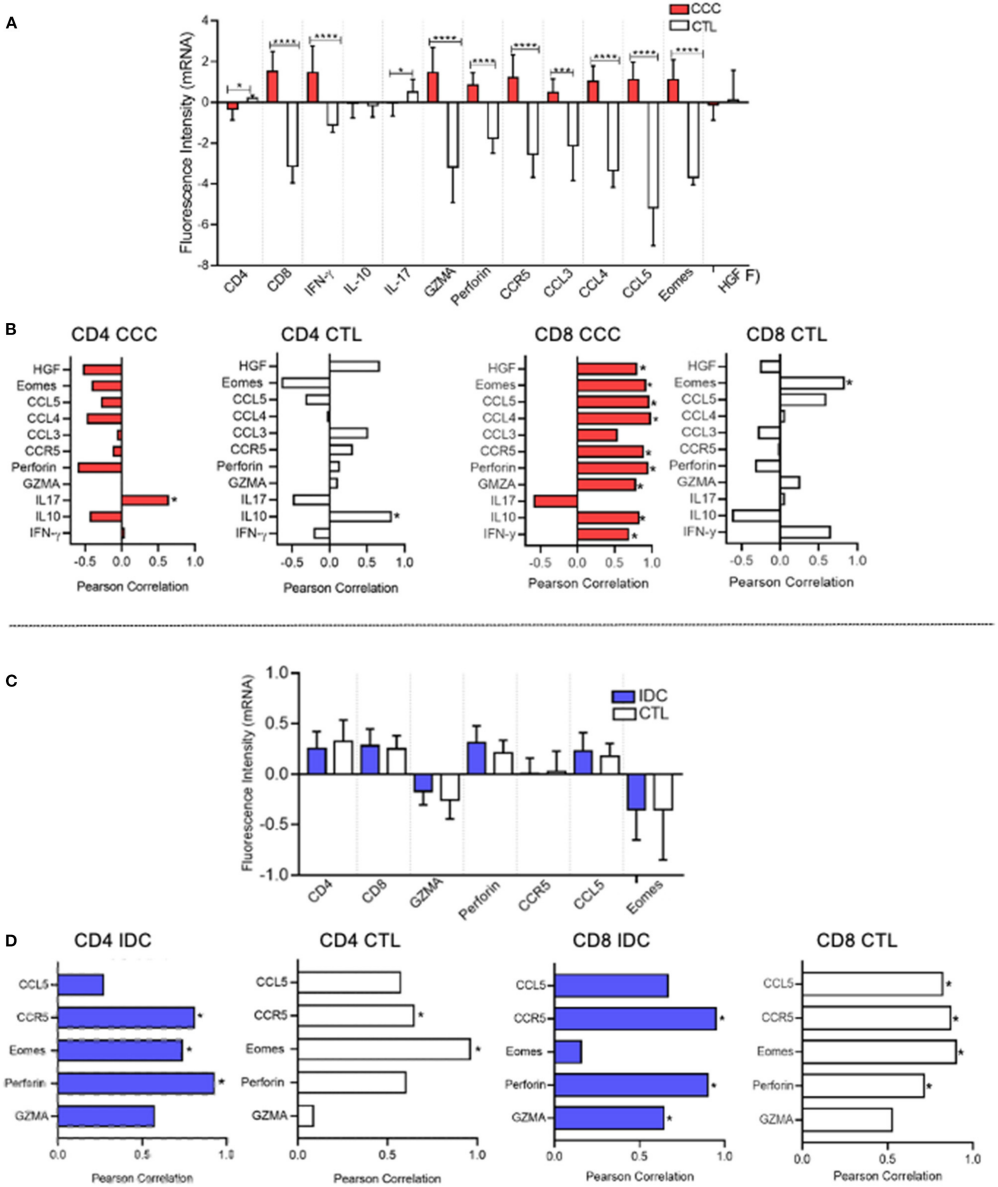
*In silico* analysis shows association between CD8 and CD4 mRNA with cytotoxic molecules in CCC and IDC hearts, respectively. **(A)** *In silico* comparative analysis between the expression of gene transcripts of molecules related to the function of CD4^+^ and CD8^+^ cells in cardiac tissue samples from patients with chronic Chagas cardiomyopathy (CCC, *n* = 10) and healthy donors (CTL, *n* = 7) **(B)** Correlation analysis between the intensity of CD8 and CD4 mRNA expression and functional molecules associated with inflammatory, regulatory, cytotoxic, and cardiotropic function in the CCC and CTL group. **(C)** Comparative analysis between the expression of gene transcripts of molecules related to the function of CD4^+^ and CD8^+^ cells in cardiac tissue samples from patients with idiopathic cardiomyopathy (IDC, *n* = 7) and Healthy donors (CTL, *n* = 8) **(D)** Correlation analysis between the intensity of CD4 and CD8 mRNA expression and functional molecules associated with inflammatory, regulatory, cytotoxic and cardiotropic function in the IDC and CTL group. Microarray data were available in Expression Omnibus databases for download [GEO- (http://www.ncbi.nlm.nih.gov/geo/)] were downloaded and subjected to statistical analysis by Graphpad Prism 8; Parametric data were analyzed by the test Student's t and non-parametric by Mann–Whitney test. Values of *p* < 0.05 were considered statistically significant. Pearson's test performed correlation analysis. *p < 0.05, *** and ****p < 0.0001.

We employed an *in silico* approach to evaluate the expression of mRNA that code for molecules related to CD4 and CD8 function in heart tissue obtained from CCC and IDC patients, using mRNA profiles available in different publicly available gene banks. Our data demonstrated up-regulation of gene transcripts for CD8, IFN-gamma, granzyme A, perforin, Eomes, CCR5, and their ligands, CCL3, CCL4, and CCL5, in cardiac tissue samples from CCC patients, compared to healthy donors (Figure 7A). Additionally, to assess the association of CD8^+^ cells with the mRNA expression intensity of the different up-regulated molecules, we used a correlation matrix, which showed an association between CD8 and HGF (cMET receptor ligand), CCR5, CCL4, CCL5, perforin, granzyme A, IL-10 and IFN-gamma in the CCC group (Figure 7B). A significant correlation was observed only between CD8 and Eomes in the control group, and no correlation of inflammatory nor cytotoxic molecules were observed with CD4 transcripts in CCC (Figure 7B).

Gene transcription profile data showed that the mRNA fluorescence intensity for the molecules CD4, CD8, Granzyme A, perforin, CCR5, CCL5, and Eomes was similar between IDC and control group (Figure 7C). Despite similar expression, the correlation analysis showed an association between CD4 and perforin in IDC but not in control (Figure 7D). Interestingly, there was also a statistically significant correlation between CD8 with CCR5 and perforin in IDC, but this was also observed in healthy individuals (Figure 7D).

## Discussion

Although both CCC and IDC are cardiomyopathies associated with high morbidity and mortality, CCC has been associated with a worse prognosis (3, 18, 42–44). While these cardiomyopathies are clinically indistinguishable, the worse prognosis observed in CCC suggests that distinct pathogenic mechanisms may be involved in the progression of these diseases. In this work, we evaluated immunological characteristics of patients with CCC or IDC and observed that CCC displays a more inflammatory systemic profile as compared to IDC. In addition, the T-cell responses, which are critical given the chronic nature of these diseases, are also quite distinct in CCC and IDC, as seen by the differential expression of immunoregulatory cytokines, cytotoxic molecules and molecules that address T-cells to the heart. Importantly, *in silico* analysis of mRNA transcripts from CCC and IDC heart tissue confirmed these differences, strengthening the hypothesis that distinct underlying immunopathology mechanisms are involved in CCC and IDC. These results point to distinct targets potentially employable as adjuvant immunotherapeutic approaches to treat these diseases.

The establishment of a predominantly systemic inflammatory immune response in individuals infected with *T. cruzi* seems to be crucial for the development of cardiac pathology (22, 29, 45–49). Here, we described elevated plasma levels of IL-6, IL-2, IL-7, IL-15, IL-17A, and IL-10 in the CCC group, as compared to IDC. In addition, dendrogram analysis showed that comparative expression of these molecules led to complete segregation between the groups. This data also reinforce the robust immune activation observed in CCC patients (50–53). Network analysis showed that IL-6, IL-2, IL-15 and IL-10 emerged as node molecules with high centrality, suggesting a broad involvement of these immune molecules in CCC. KEGG enrichment analyses identified inflammatory pathways as the main ones amongst the first 20 pathways with lowest FDR, of particular interest, the T-cell signaling and the TNF signaling pathways. These data corroborate previous studies that highlight the role of inflammatory molecules in the immunopathology of Chagas disease (54–56). Exploiting the molecules that compose these pathways may provide potential targets to treat CCC.

Given that T-cells are central in the adaptive immune response observed in chronic diseases, including CCC and IDC (30, 32, 33, 57–61), we proposed to evaluate the functional characteristics of different T-cell subpopulations present in the peripheral blood of CCC and IDC patients. Our results suggest that while CD8^+^ T-cells are the main ones associated with the immunoregulatory potential in CCC, CD4^+^ T-cells are associated with IDC. CD8^+^ cells display increased frequency of expression of TNFR1, TNF, IL-10, and IL-17 when compared to IDC, and CD4^+^ T-cells display increased expression of TNF and TNFR1 in IDC as compared to CCC. Increase expression of cytokines with different functional profiles observed in CCC is possibly related to a control mechanism for the intense inflammation observed in those patients (53, 62–66). Analysis of the ratio TNFR1/IL-10R revealed a predominantly inflammatory profile in CD8^+^ T-cells from CCC compared to IDC. A similar tendency was observed in the CD4^+^ T-cells from IDC, although not statistically significant. Previous studies have shown that CD8^+^ T cells expressing TNF are indeed the predominant cell type in the hearts of CCC patients (45), and that CD4^+^ T cells expressing activation molecules are present in the heart of IDC patients, although not increased in relation to other leukocytes (67). Of note, the TNF signaling pathway appeared as one of the key pathways associated with the altered cytokine profile in CCC, strengthening the role of this molecule in CCC and suggesting this pathway as a potential intervention target.

T-cell cytotoxic function is essential for resistance to *T. cruzi* infection through the lysis of infected cells (68–70), and may also be important in IDC where viral infection may have played a role (17, 71, 72). However, uncontrolled T-cell mediated cytotoxicity may contribute to cardiac injury and dysfunction (45, 73, 74). To determine the cytotoxic potential of the different cell populations, we evaluated the expression of Eomes, a critical transcription factor for the differentiation of activated CD8^+^ T-cells and for controlling expression of inflammatory cytokines, and the cytotoxic molecules granzyme A and perforin (75). It is noteworthy to mention that expression of most cytotoxic molecules was similar between groups in all T-cell populations. Thus, these cells may potentially be related to pathology in both diseases. However, we observed a high expression of Eomes by CD8^+^ T-cells in CCC, reinforcing the previously suggested role for these cells in CCC pathology (45).

While our data also evidenced the effector capacity of T *helper* lymphocytes in CCC, shown by the increased frequency of expression of granzyme A in CD4^+^ T-cells, as compared to IDC, the presence of cytotoxic CD4^+^ T-cells in the hearts of CCC has not been investigated. These cells recognize antigenic peptides via MHC class II and are capable of secreting cytolytic granules that can induce apoptosis of target cells (76, 77). Thus, it is valid to speculate that CD4^+^ T-cells may also contribute to the immunopathology in CCC. Favoring this hypothesis, pro-inflammatory and potentially effector CD4^+^ T-cells have been associated with pathology in experimental models of *T. cruzi* infection, particularly in the absence of regulatory B-cells (78).

To determine the underlying mechanism related to the recruitment of these different T-cell populations to the heart tissue of CCC or IDC patients, we first evaluated the expression of the chemotactic receptors CCR5 and cMET, which display cardio tropic activity (79). In addition, cytolytic CD8^+^ T-cells expressing cMET have been associated with increased intracellular levels of inflammatory cytokines such as IFN-gamma and TNF (80). The upregulation of CCR5 in CD8^+^ T-cells from *T. cruzi*-infected mice has been associated with tissue damage (81, 82). Genetic variants of CCR5 have been associated with human Chagas disease (83), and high CCR5 expression by leukocytes has also been associated with severe CCC (27, 84). The analysis of CCR5 expression frequency showed a high frequency of CD4^+^, CD8^+^ and TCRgamma-delta+ DN T-cells expressing this molecule in CCC compared to IDC. Moreover, a positive correlation between the frequency of CD8^+^ CCR5^+^ and CCL4, a ligand for CCR5, was observed in CCC but not IDC. The high frequency of cMET^+^ cells in the CCC group compared to IDC, as well as the association between CD8^+^cMET^+^CCR5^+^ T-cells with the frequency of CD8^+^IFN-gamma^+^ and CD8^+^EOMES^+^ T-cells only in the CCC patient group, strongly suggest that these molecules mediate the recruitment of inflammatory and cytotoxic CD8^+^ T-cells to cardiac tissue in CCC. Our data from the *in silico* analysis support this hypothesis, since it revealed an increase in the intensity of CD8 transcripts in the cardiac tissue of CCC patients compared to healthy donors. Furthermore, there was a correlation between the intensity of expression of CD8 mRNA and molecules with inflammatory potential (IFN-gamma), cytotoxic (Eomes, granzyme A, perforin), regulatory (IL-10), and cardiotropic (CCR5, CCL4, and CCL5 and HGF) in CCC but not IDC. Importantly, previous studies in experimental models of *T. cruzi* infection have shown that treatment with Met-RANTES, a CCR5 antagonist, ameliorated the cardiac tissue damage observed in treated animals (85). Thus, controlling the recruitment of potentially cytotoxic CCR5^+^CD8^+^ T-cells may emerge as a possible immunotherapeutic strategy in CCC.

The increased expression of CCR4, which has been related to T-cell infiltration in heart inflammatory conditions in a murine model (79, 86), and CXCR3^+^ CCR4^+^ cells by CD4^+^ T-cells in IDC implicates these molecules in the recruitment of this cell subpopulation to the heart, revealing distinct cell recruitment mechanisms in CCC and IDC. In addition, gamma-delta+ DN T-cells also express higher CXCR3 receptors in IDC as compared to CCC, suggesting a potential role for these cells in IDC (87–90). *In silico* analysis showed an association of CD4 with perforin in IDC, which was not observed in healthy hearts, adding to the hypothesis that these cells are involved in tissue pathology in IDC. The identification of TCRgamma-delta+ DN T-cells was not possible in this analysis since this can only be determined using multiparameter immunostaining *in situ*, or single cell analysis, due to the need to exclude CD4 and CD8 expression in the gamma-delta+ T-cells. The use of a small molecule CXCR3 receptor antagonist has been shown to improve cardiac remodeling (91) in experimental models, opening perspectives for studying its use in human cardiomyopathies, where this molecule may play a role, such as in IDC.

CXCR3 and CCR4 expression have been shown to be induced by cMET, which has cardiotropic effects (79). Although the expression of this molecule is low in IDC, it is possible that the observed levels are sufficient to induce these chemokine receptors. We also observed an association of CD8^+^ T-cells and cMET in CCC. Thus, it is tempting to hypothesize that, despite the involvement of distinct T-cell populations in IDC and CCC, control of cMET expression (or its ligand, HGF), may emerge as a strategy to control T-cell recruitment in IDC and CCC. Specific inhibition of cMET has shown promising effects to treat several types of cancer (92). However, given that the HGF/cMET axis is important in heart function (93), more in-depth studies need to be performed to evaluate this possibility.

## Limitations Of The Study

Despite dealing with clinically similar cardiomyopathies, it is important to bear in mind that these diseases develop under a distinct time scale, which may impact the differential immune responses observed. It is, however, difficult to determine exactly when disease started, posing a limitation as to the role of time to disease development and intensity of the observed immune response. In addition, while the pathology associated with both diseases may develop over years, CCC results from an acquired infection, not the same in IDC. This difference in etiology may also influence the inflammatory response, potentially explaining the exuberant response observed in CCC. The results shown in this study significantly add to the current literature as they represent, to the best of our knowledge, the first comparison of cellular and systemic immune response between CCC and IDC, two deadly cardiomyopathies. In addition to bringing insights to the mechanisms of pathology underlying these diseases, our studies point to potential targets that may be applied to control the immune response and, thus, pathology in these diseases. However, further studies need to be developed to validate these potential strategies and instruct clinical practice, which were not within the scope of present study.

## Conclusion And Future Directions

In conclusion, the data from the comparative analysis between the different cardiomyopathies that manifest clinically as heart failure showed that CCC is associated with a strong immune activation, with a soluble cytokine signature that distinguishes it from IDC. The *ex vivo* analysis of peripheral T-cell responses, involving cytokine and cytotoxic molecule expression, and the *in silico* analysis of cardiac tissue suggest that inflammatory and cytotoxic CD8^+^ T-cells are strongly associated with CCC, while CD4^+^ T-cells are associated with IDC. Furthermore, differential expression of chemotactic receptors by CD4^+^ and CD8^+^ T-cells, suggest distinct mechanisms of recruitment to the heart in IDC and CCC. Our data demonstrated clear immunological differences in otherwise clinically similar cardiomyopathies, clarifying mechanisms of immunopathology and pointing to potential targets for immune-mediated intervention in these diseases. Our data demonstrate clear immunological differences in clinically similar cardiomyopathies, elucidating the involvement of molecules potentially involved in the immunopathology of these diseases and pointing to potential targets for immunity-mediated intervention. Future studies, using modulators of chemokine receptor function and TNF-mediated inflammatory responses, are underway in our laboratory to validate whether the modulation of cellular recruitment and inflammatory responses will have a direct translational application in CCC and IDC.

## Data Availability Statement

The original contributions presented in the study are included in the article/Supplementary Material, further inquiries can be directed to the corresponding author/s.

## Ethics Statement

The studies involving human participants were reviewed and approved by COEP-UFMG/CONEP. The patients/participants provided their written informed consent to participate in this study.

## Author Contributions

EN and CK performed all cell processing and cultures, as well as FACS analysis. AS, TV, FV, and JC aided in sample collection and blood processing. TS-S performed *in silico* analyses. LP performed the network and KEEG analyses. MN was responsible for clinical care, characterization, and for overlooking material collection from all patients. CB aided in experimental design and provided the Eomes antibody used in initial experiments. AT was responsible for the Bioplex assays. KG contributed to experimental design and data analysis. WD designed the studies and overlooked all experiments and data analysis. All authors contributed to writing and reviewing the manuscript.

## Funding

This work was supported by FAPEMIG (#Universal 2014), CNPq (#Universal 2015), INCT-DT, and NIAID-NIH (1R01AI138230-01). CK, MN, TS-S, CB, KG, and WD are CNPq fellows and EGAN is a CAPES fellow.

## Conflict of Interest

The authors declare that the research was conducted in the absence of any commercial or financial relationships that could be construed as a potential conflict of interest. The reviewer JS declared a shared affiliation with one of the authors AT, to the handling editor at time of review.

## Publisher's Note

All claims expressed in this article are solely those of the authors and do not necessarily represent those of their affiliated organizations, or those of the publisher, the editors and the reviewers. Any product that may be evaluated in this article, or claim that may be made by its manufacturer, is not guaranteed or endorsed by the publisher.
