# Supplementary figures and images for: T-Cell Subpopulations Exhibit Distinct Recruitment Potential, Immunoregulatory Profile and Functional Characteristics in Chagas versus Idiopathic Dilated Cardiomyopathies
Source: Front Cardiovasc Med. 2022 Feb 2;9:787423. doi: 10.3389/fcvm.2022.787423 (PMC8847602; doi:10.3389/fcvm.2022.787423)

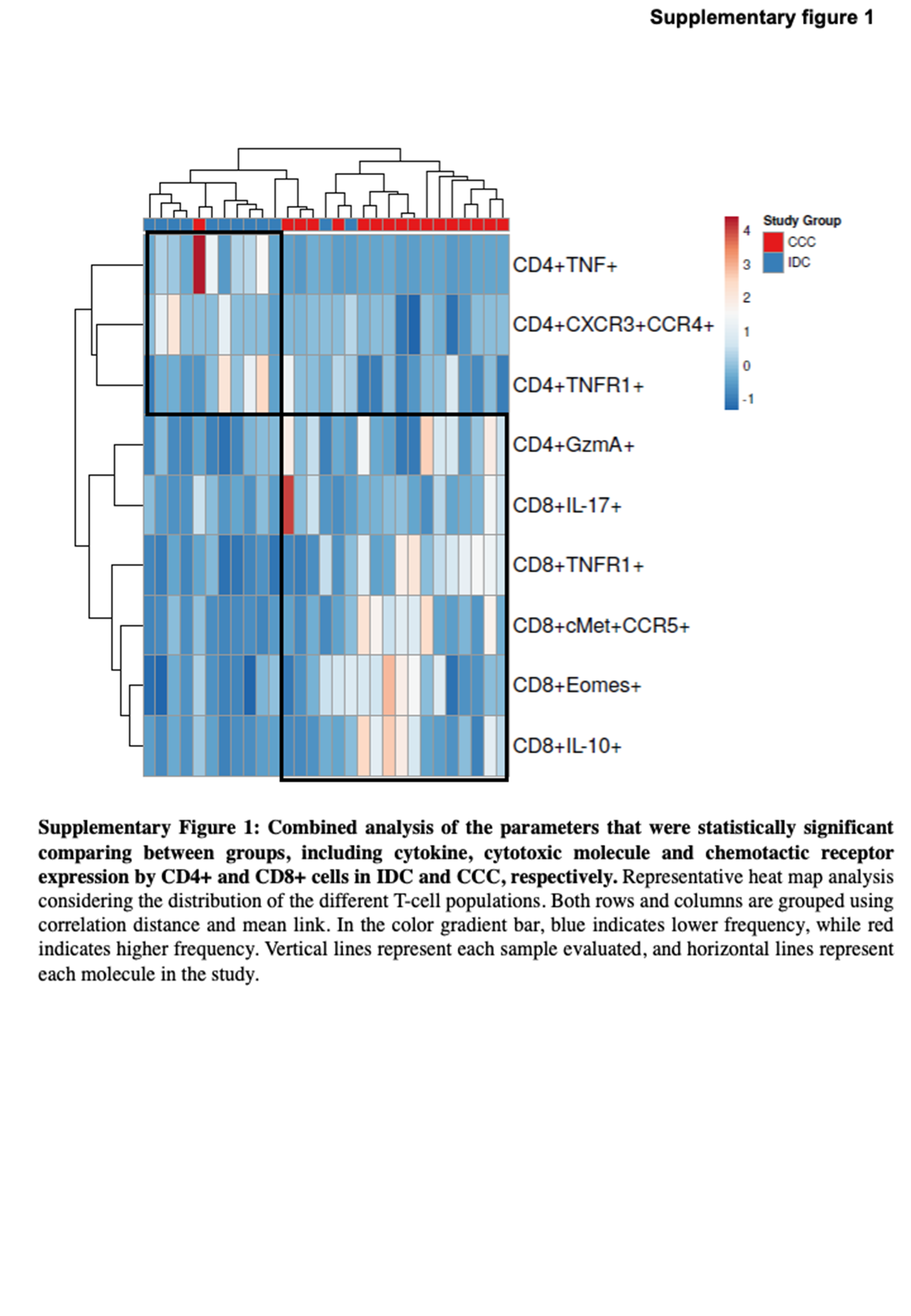

Supplement: Supplementary file 1 [file Image_1.TIF]
